# Activin A signaling stimulates neutrophil activation and macrophage migration in pancreatitis

**DOI:** 10.1038/s41598-024-60065-y

**Published:** 2024-04-23

**Authors:** Mark B. Wiley, Jessica Bauer, Valentina Alvarez, Kunaal Mehrotra, Wenxuan Cheng, Zoe Kolics, Michael Giarrizzo, Komala Ingle, Agnieszka B. Bialkowska, Barbara Jung

**Affiliations:** 1 Department of Medicine, University of Washington, 1959 NE Pacific Street, Seattle, WA 98195 USA; 2 Department of Biochemistry, University of Washington, Seattle, WA 98195 USA; 3 Department of Medicine, Renaissance School of Medicine at Stony Brook University, Stony Brook, NY 11794 USA

**Keywords:** Activin A, Acute pancreatitis, Chronic pancreatitis, Digital spatial profiling, Cerulein, Acute pancreatitis, Autoimmune diseases

## Abstract

Acute Pancreatitis (AP) is associated with high mortality and current treatment options are limited to supportive care. We found that blockade of activin A (activin) in mice improves outcomes in two murine models of AP. To test the hypothesis that activin is produced early in response to pancreatitis and is maintained throughout disease progression to stimulate immune cells, we first performed digital spatial profiling (DSP) of human chronic pancreatitis (CP) patient tissue. Then, transwell migration assays using RAW264.7 mouse macrophages and qPCR analysis of “neutrophil-like” HL-60 cells were used for functional correlation. Immunofluorescence and western blots on cerulein-induced pancreatitis samples from pancreatic acinar cell-specific *Kras* knock-in (*Ptf1aCre*^*ER*^*™; LSL-Kras*^*G12D*^) and functional WT *Ptf1aCre*^*ER*^*™* mouse lines mimicking AP and CP to allow for in vivo confirmation. Our data suggest activin promotes neutrophil and macrophage activation both in situ and in vitro, while pancreatic activin production is increased as early as 1 h in response to pancreatitis and is maintained throughout CP in vivo. Taken together, activin is produced early in response to pancreatitis and is maintained throughout disease progression to promote neutrophil and macrophage activation.

## Introduction

Acute pancreatitis (AP), inflammation of the pancreas leading to the destruction of acinar cells, has increased by 64% in global incidence between 1990 and 2019^1^. Furthermore, AP-related healthcare expenses exceed $2.5 billion annually in the U.S.^2,3^. Despite the increase in the incidence of AP, research on pancreatitis has decreased more than any other gastrointestinal (GI) disease over 50 years, with significant issues surrounding patient recruitment in clinical studies^4,5^. Current treatment options are limited to supportive care, with mortality rates exceeding 30% in severe cases^6,7^. Following recovery from AP, approximately 17% of AP patients develop recurrent pancreatitis, and an additional 8% progress into chronic pancreatitis (CP)^8^. The risk for the development of pancreatic ductal adenocarcinoma (PDAC) is three to ten times higher following AP which increases with the number of times the patient experiences AP^9^. The most significant risk of PDAC development is observed in AP patients with underlying CP^9^, highlighting the need to identify treatment options early in the progression of AP to CP to PDAC.

Important work has highlighted the potential for targeting trypsinogen and other proteases in AP^10,11^. However, multiple human trials have failed to show the benefits of trypsinogen inhibition^12^. Similarly, direct targeting of the immune system via exogenous administration of IL-10 has demonstrated no benefit for endoscopic retrograde cholangiopancreatography (ERCP) induced pancreatitis, and studies centered on alternative targets are lacking^13^. Due to the lack of molecular targets, several studies have attempted to improve outcomes in AP via naturopathic approaches, including timing and volume of fluid resuscitation. Recently, the WATERFALL clinical trial was terminated early because it found no improvement in clinical outcomes in patients receiving early fluid resuscitation, and it put the patients at an increased risk for fluid overload^14^. Evidence show that early administration of lactated ringer’s solution instead of saline reduces systemic inflammation and improves outcomes in AP patients. However, larger cohorts are necessary to confirm these findings^15,16^.

Encouraging results have been observed in regulating Ca^2+^ channels via ORAI1 inhibition, the primary store-operated Ca^2+^ entry channel on pancreatic acinar cells^17,18^. This led to the phase II open-label clinical trial for Auxora, which found that patients who received Auxora better tolerate solid foods and have a reduced systemic inflammatory response syndrome^19^. This ongoing clinical trial is also being investigated as a potential therapeutic for recurrent AP and CP^20^. Additionally, premature release of pancreatic enzymes leads to excessive visceral adipose lipolysis and release of fatty acids into the microenvironment^21^. These lipids contribute to macrophage activation^22^, and studies have found that dysregulated lipolysis significantly contributes to AP severity^23^. These encouraging findings require further investigation to determine if targeting pancreatic lipase improves clinical outcomes in AP patients.

Our group has found promising results through the inhibition of activin A (activin), a transforming growth factor β (TGFβ) superfamily member, which plays a critical role in inflammation, cancer, and induction of other cytokines^24–26^. We previously reported increased serum levels of activin in AP patients compared to healthy controls^26^, an effect independent of body mass index (BMI)^27^. Furthermore, high activin levels at admission predicted more extended hospital stay than intermediate or low activin levels^28^. Anti-activin-neutralizing antibody treatment in a cerulein-induced murine model of AP reduced disease severity and neutrophil infiltration into the pancreas^27^. This same treatment effectively reduced mortality in a more severe mouse model of AP in which *ob/ob* mice received injections of IL-12/IL-18 to induce AP^28^.

Several exocrine pancreas cells produce activin and its production increases in response to insults. Immortalized human pancreatic ductal cells contain the mRNA necessary to produce activin, which is upregulated in response to treatment with tumor necrosis factor α (TNFα)^27^. Under homeostatic conditions, the production of activin in acinar cells is minimal. However, a significant increase in production has been observed in response to cerulein and in acinar-to-ductal metaplasia (ADM)^29^. Pancreatic stellate cells also produce substantial quantities of activin following activation^27^. Macrophages produce activin, and activin stimulation of macrophages increases mRNA production of TNFα and IL-1β in vitro^27,30^. Furthermore, TNFα stimulates neutrophil release of activin^31^. These findings suggest that nearly all cells involved in pancreatitis and PDAC development produce activin, underscoring its pivotal role in the pathogenesis of these conditions.

Building on the published data and activin’s role in several inflammatory conditions, including inflammatory bowel disease^24^, asthma^25^, viral infections^32^, and obesity^33^, we formulated a hypothesis. We proposed that activin is produced early in AP to perpetuate local inflammation and is maintained throughout disease progression. To test this hypothesis, we employed digital spatial profiling (DSP, NanoString Technologies) on a human chronic pancreatitis (CP) tissue sample to identify cellular signatures relative to activin co-localization in pancreatitis. We also used real-time quantitative polymerase chain reaction (qPCR) on neutrophil-like HL-60 cells to confirm our DSP findings. We utilized two distinct mouse models to determine the local production of activin in both acute and chronic inflammation-assisted PDAC in vivo, coupled with in vitro transwell migration assays in the presence/absence of anti-activin neutralizing antibodies to investigate activin’s ability to stimulate immune cell migration. This comprehensive approach allowed us to test the hypothesis that activin is produced early in response to pancreatitis, thereby driving immune cell activation and potentially serving as a therapeutic target.

## Results

### Digital spatial profiling separates tissue based on activin co-localization

**Figure 1:**
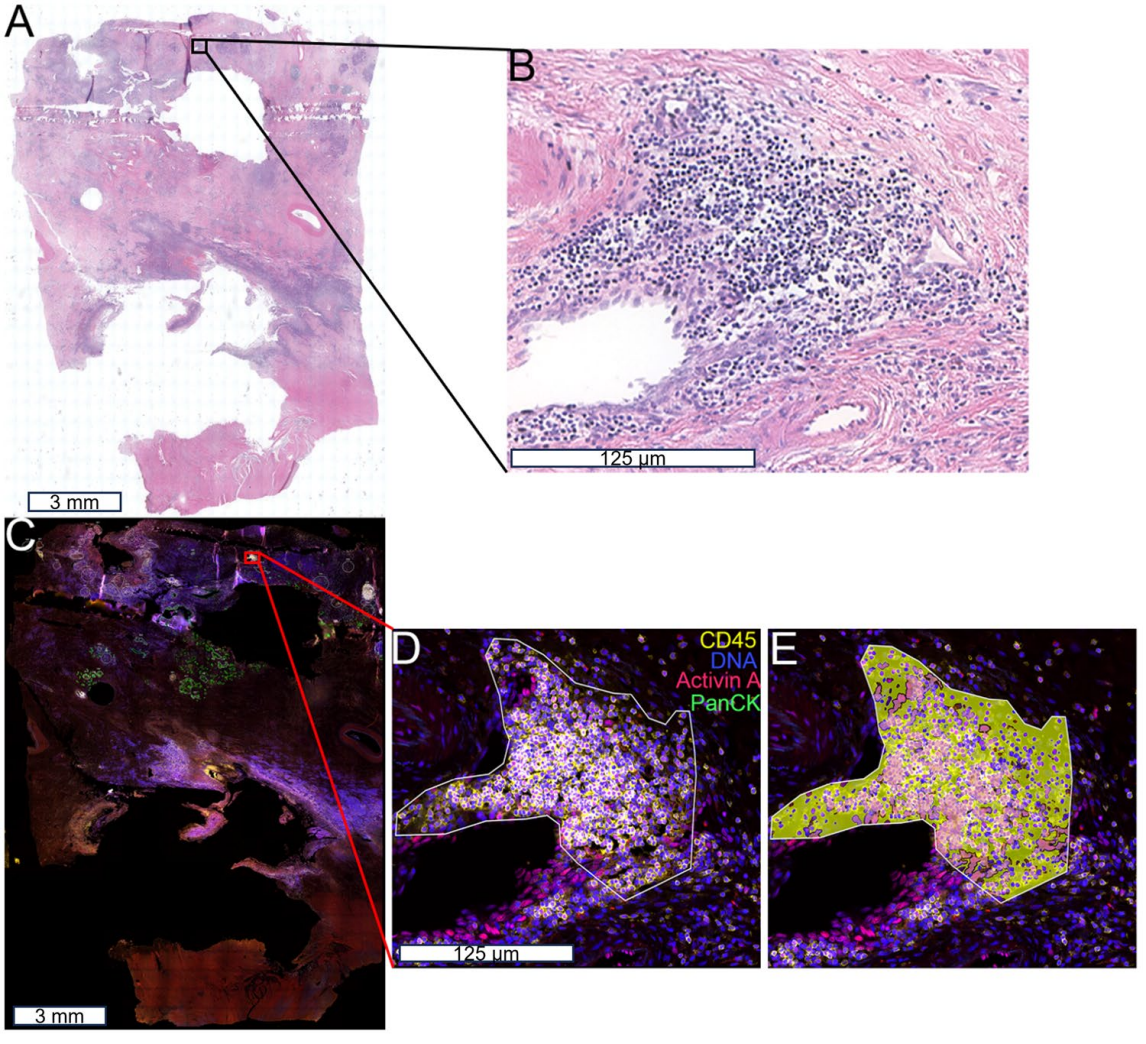
DSP technology permits the separation of distinct tissue compartments in the human pancreas. (**A**,**B**) Hematoxylin and Eosin staining was performed on a human chronic pancreatitis tissue sample, which was used as a reference for (**C**) the DSP staining. (**D**) ROIs were selected based on localization and expression of four fluorescent markers (PanCK, green; DNA, blue; CD45, yellow; and activin, red) (**E**), which were separated into activin ( +) and ( −) AOIs. ROIs were selected in areas with high activin and CD45 expression and no PanCK expression to avoid areas undergoing acinar-to-ductal metaplasia.

To visualize the location of inflammatory clusters within a human chronic pancreatitis tissue sample, we first performed H&E staining (Fig. 1A,B). This stain also permitted us to specifically choose regions of the tissue for DSP analysis without acinar-to-ductal metaplasia (ADM) or PanIN lesions. To determine the cellular signatures associated with activin co-localization in pancreatitis, we performed DSP analysis on a serial slice from the same chronic pancreatitis patient with activin included as a fluorescent marker (Fig. 1C–E). Regions of interest (ROIs) were selected where high expression of activin and CD45 were observed with no expression of PanCK to identify clusters of inflamed tissue that were not undergoing ADM (Fig. 1D). These ROIs were then further separated into activin ( +) (pink) and activin ( −) (yellow) areas of illumination (AOIs) (Fig. 1E). This approach permitted the separation of tissue compartments based on activin co-localization and quantifying 57 proteins of interest relative to activin co-localization.

### Activin-dependent signaling compartments are present in patient sample of chronic pancreatitis

**Figure 2:**
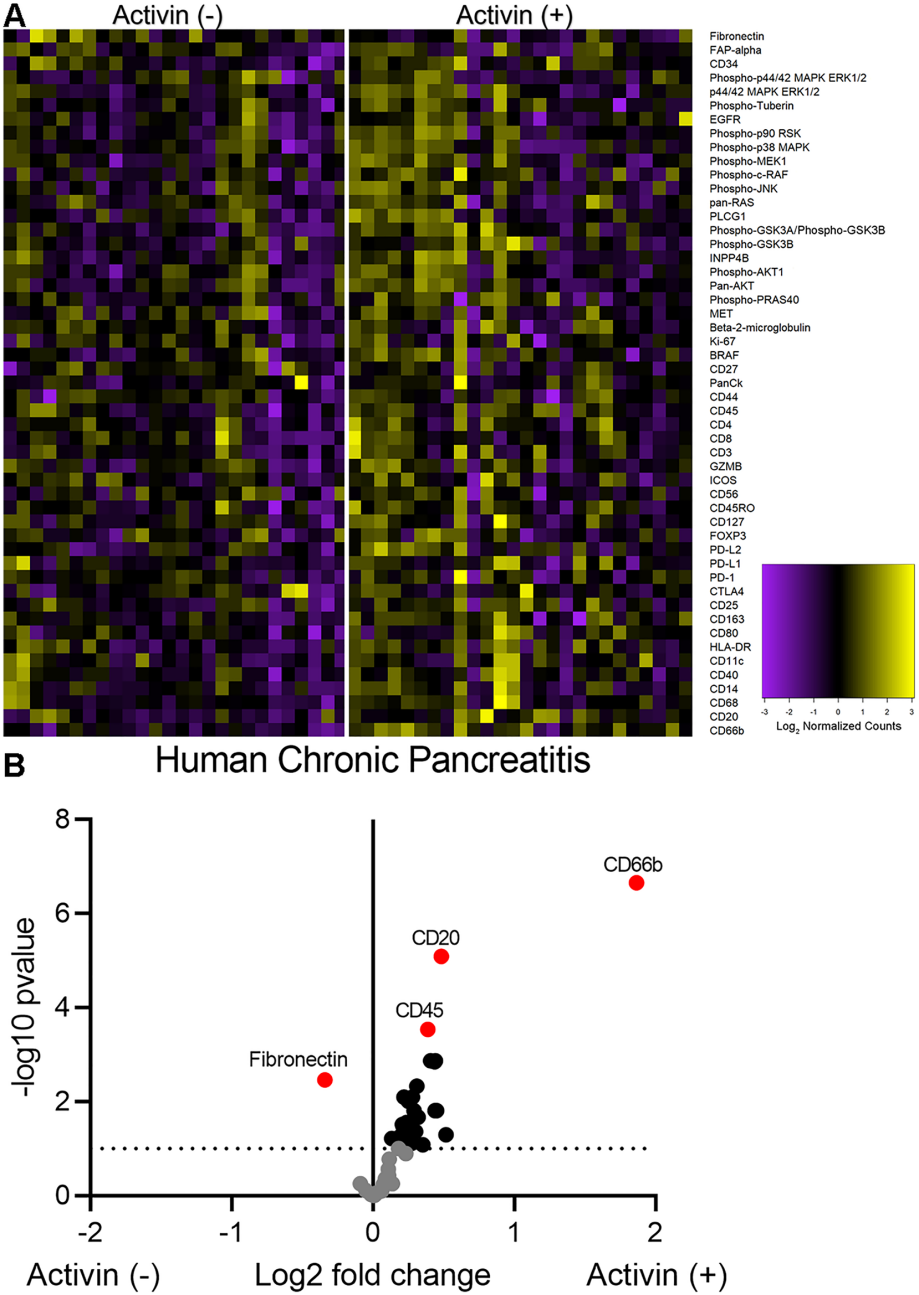
Distinct signaling compartments that were activin-dependent were identified via DSP analysis. Regions of Interest (ROIs) were selected in areas where dense expression of CD45 was observed and barcode collection occurred in areas of illumination (AOIs) based upon activin co-localization. This permitted quantifcation of several proteins relative to activin co-localization in situ. (**A**) Activin ( +) areas (right) show the highest heat signature for several markers of immune cell activation compared to activin ( −) areas (left). A total of 52 ROIs were identified which were separated into 26 activin ( −) and 26 activin ( −) AOIs for quantification and comparison. Each column represents one AOI and 26 AOIs were investigated across activin ( +) and ( −) compartments. (**B**) Volcano plot indicating that several proteins, including CD66b, CD20, CD45, and Fibronectin, are significantly differentially expressed across activin co-localization in pancreatitis. Data expressed as log2 normalized counts, n = 26.

Innate immune cells, including neutrophils and macrophages, produce activin, which can signal in an autocrine/paracrine fashion to promote pro-inflammatory phenotypes of these cells^27,31,34^. Additionally, activin can signal along the MAPK and PI3K pathways. Therefore, we included several markers of the MAPK, PI3K, and immune cells in our DSP panel of quantifiable proteins (Supplementary Table 1). Several distinct signaling patterns were observed that appeared to be dependent upon activin co-localization in pancreatitis (Fig. 2A). Additionally, several proteins were found to be significantly upregulated in activin ( +) AOIs when compared to activin (−) AOIs in pancreatitis (Fig. 2B). The proteins that displayed a significant change in expression across activin co-localization included CD66b, CD20, CD45, CD11c, HLA-DR, PD-L2, Fibronectin, CD68, phospho-p90 RSK (T359/S363), PanCK, CD14, p44/42 MAPK ERK 1/2, phospho-c-RAF (S388), phospho-JNK (T183/Y185), phospho-GSK3a (S21), CD80, CD45RO, CD34, CD4, INPP4B, phospho-MEK1 (S217/S221), CD3, PLCG1, phospho-GSK3b (S9), BRAF, CD163, GZMB, CD127, and CD25. These data suggest that activin stimulates the upregulation of several inflammatory markers, PI3K pathway markers, and MAPK pathway markers included in our DSP panel.

### Activin ( +) AOIs display increased macrophage and activated neutrophil infiltration

**Figure 3:**
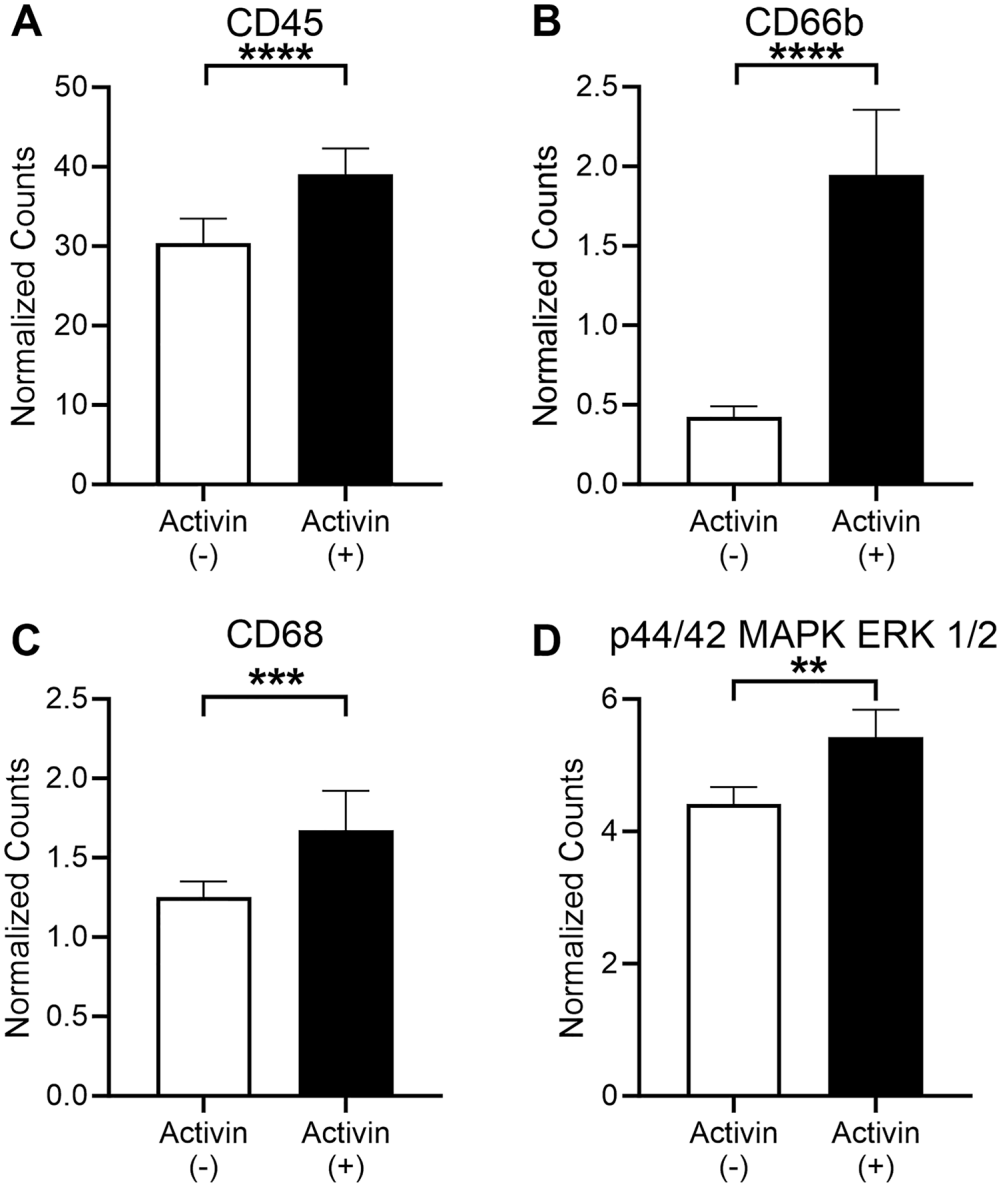
Activin ( +) AOIs display increased markers of neutrophil activation and markers of macrophages. Activin ( +) AOIs showed significant increases in (**A**) CD45, (**B**) CD66b, (**C**) CD68, and (**D**) p44/42 MAPK ERK 1/2. Data was analyzed via Linear Mixed Modeling with Benjamini–Hochberg multiple-correction test (**p < 0.01, ***p < 0.001, ****p < 0.0001, n = 26).

We previously reported that anti-activin intervention in a mouse model of AP reduces neutrophil infiltration into the pancreas^27^. Therefore, we included several markers of immune cells and markers of effector cells in our DSP panel to determine if activin co-localization is associated with these cells’ recruitment and/or activation. In the human CP tissue sample, we observed a significant increase in activin ( +) AOIs when compared to activin ( −) AOIs in CD45 (( +): 39.01 normalized counts ± 3.31 S.E.M., ( −): 30.40 normalized counts ± 3.09 S.E.M.) (Fig. 3A), CD66b (( +): 1.94 normalized counts ± 0.41 S.E.M., ( −): 0.42 normalized counts ± 0.07 S.E.M.) (Fig. 3B), CD68 (( +): 1.67 normalized counts ± 0.25 S.E.M., ( −): 1.25 normalized counts ± 0.10 S.E.M.) (Fig. 3C), and p44/42 MAPK ERK 1/2 (( +): 5.43 normalized counts ± 0.41 S.E.M., ( −): 4.41 normalized counts ± 0.26 S.E.M.) (Fig. 3D) (n = 26 for all AOIs). These data suggest that activin may be responsible for increased total immune cell (CD45) recruitment, neutrophil activation (CD66b), macrophage migration (CD68), and MAPK activation (p44/42 MAPK ERK 1/2) in the inflamed pancreas.

### Activin stimulates macrophage migration and neutrophil activation in vitro

**Figure 4:**
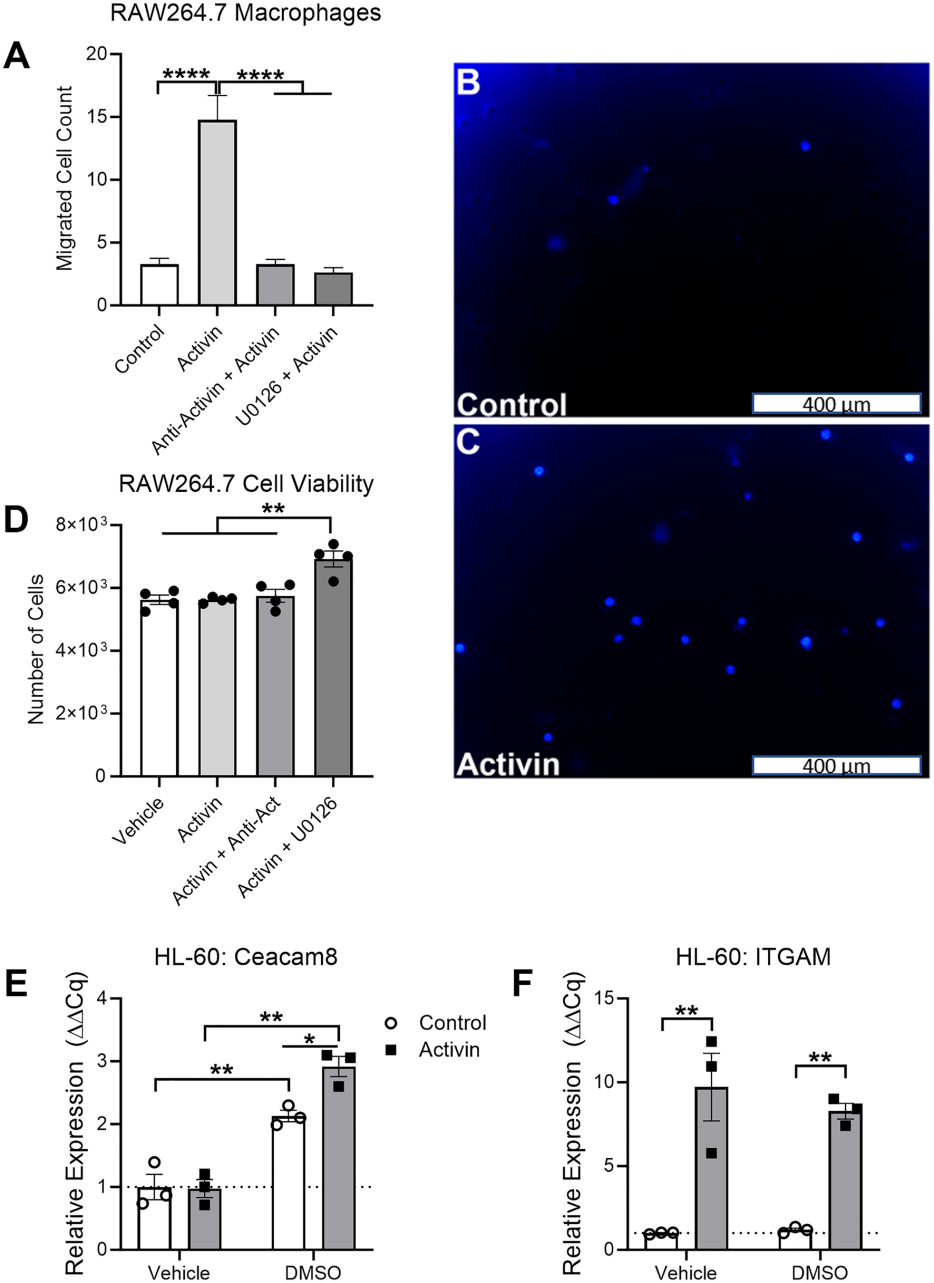
Activin stimulates macrophage migration and neutrophil activation in vitro. (**A**) Treatment of RAW264.7 macrophages with activin increases the migratory capacity of these cells which is ablated by anti-activin or MAPK inhibiton in vitro. (**B**,**C**) Representative images of transwells from the migration assay which display an increase in migrated cells in activin stimulated samples. (**D**) A cell viability assay was performed on macrophages to confirm that the trasnwell migration assay results were not due to changes in cell viability. (**E**) Stimulation of differentiated HL-60 cells with activin further increases CD66b production. (**F**) Activin increases production of the gene encoding CD11b in HL-60 cells regardless of DMSO exposure. Data was analyzed via regular one-way ANOVA with Tukey multiple comparison’s test (*p < 0.05, **p < 0.01, ***p < 0.001, ****p < 0.0001, n = 3 or 5).

We performed several in vitro experiments on RAW264.7 macrophages and HL-60 cells to confirm our DSP findings. We first utilized transwell migration assays with activin as a pre-treatment to determine if activin primed immune cells for migration and invasion into the pancreas. We found that stimulation of RAW264.7 macrophages with activin increases their migratory capacity (14.76 migrated cells ± 1.96 S.E.M., n = 5) when compared to vehicle-treated cells (3.28 migrated cells ± 0.47 S.E.M., n = 5). The transwell migration assay was also performed on RAW264.7 macrophages that received anti-activin neutralizing antibodies with activin (3.28 migrated cells ± 0.39 S.E.M., n = 5), which ablated the activin-induced migration. Given our data in Fig. 3 suggesting that activin co-localization is associated with increased MAPK activation, we also performed a transwell migration assay on RAW264.7 macrophages pre-treated with the MEK-inhibitor U0126 and activin, which displayed a significant reduction in migratory capacity (2.64 migrated cells ± 0.36 S.E.M., n = 5) (Fig. 4A). We also provided zoomed-in (40 ×) representative images of our control and activin conditions from the transwell migration assays to provide further visual evidence of the activin-induced increase in the migratory capacity of macrophages in vitro (Fig. 4B,C). Additionally, we performed a cell viability assay to confirm that the results from the transwell migration assays were due to increased migratory capacity and not to cytotoxic conditions. We found no change in cell viability across control (5.3 × 10^3^ cells ± 150 S.E.M., n = 4), activin (5.6 × 10^3^ cells ± 45 S.E.M., n = 4), or anti-activin + activin (5.8 × 10^3^ cells ± 202 S.E.M., n = 4) conditions. We did observe a significant increase in the number of cells in the U0126 + activin treated cells (6.9 × 10^3^ cells ± 252 S.E.M., n = 4), suggesting that inhibition of MEK may lead to the proliferation of these cells. Despite the increase in cell numbers, there was no observed increase in migrated cells in the U0126 treated cells, confirming that the effects observed in Fig. 4A were due to increased migratory capacity (Fig. 4D). These data suggest that activin stimulates macrophage migration in an activin- and MAPK-dependent manner. Next, we wanted to confirm that the increase in CD66b expression in activin ( +) AOIs was due to activin stimulation of neutrophils. Therefore, we employed the HL-60 cell line and stimulated them to differentiate into neutrophil-like cells with DMSO as previously described^35–37^. We found that expression of the gene encoding CD66b (Ceacam8) was significantly increased in HL-60 cells exposed to DMSO for 24 h (2.13 relative expression units ± 0.09 S.E.M., n = 3) when compared to control conditions (1.00 relative expression units ± 0.20 S.E.M., n = 3) which was further increased via activin stimulation (2.92 relative expression units ± 0.16 S.E.M., n = 3) (Fig. 4E). Interestingly, we observed a significant increase in the gene encoding CD11b (ITGAM) in activin stimulated HL-60 cells without DMSO (9.71 relative expression units ± 2.02 S.E.M., n = 3) and with DMSO (8.28 relative expression units ± 0.47 S.E.M., n = 3) when compared to control conditions in the absence (1.00 relative expression units ± 0.03 S.E.M., n = 3) and presence (1.19 relative expression units ± 0.11 S.E.M., n = 3) of DMSO (Fig. 4F). These data suggest activin stimulates macrophage migration via the MAPK pathway and promotes neutrophil activation which may further perpetuate AP progression.

### Activin production is increased in a murine model of AP, which is further increased in inflammation-assisted PDAC

**Figure 5:**
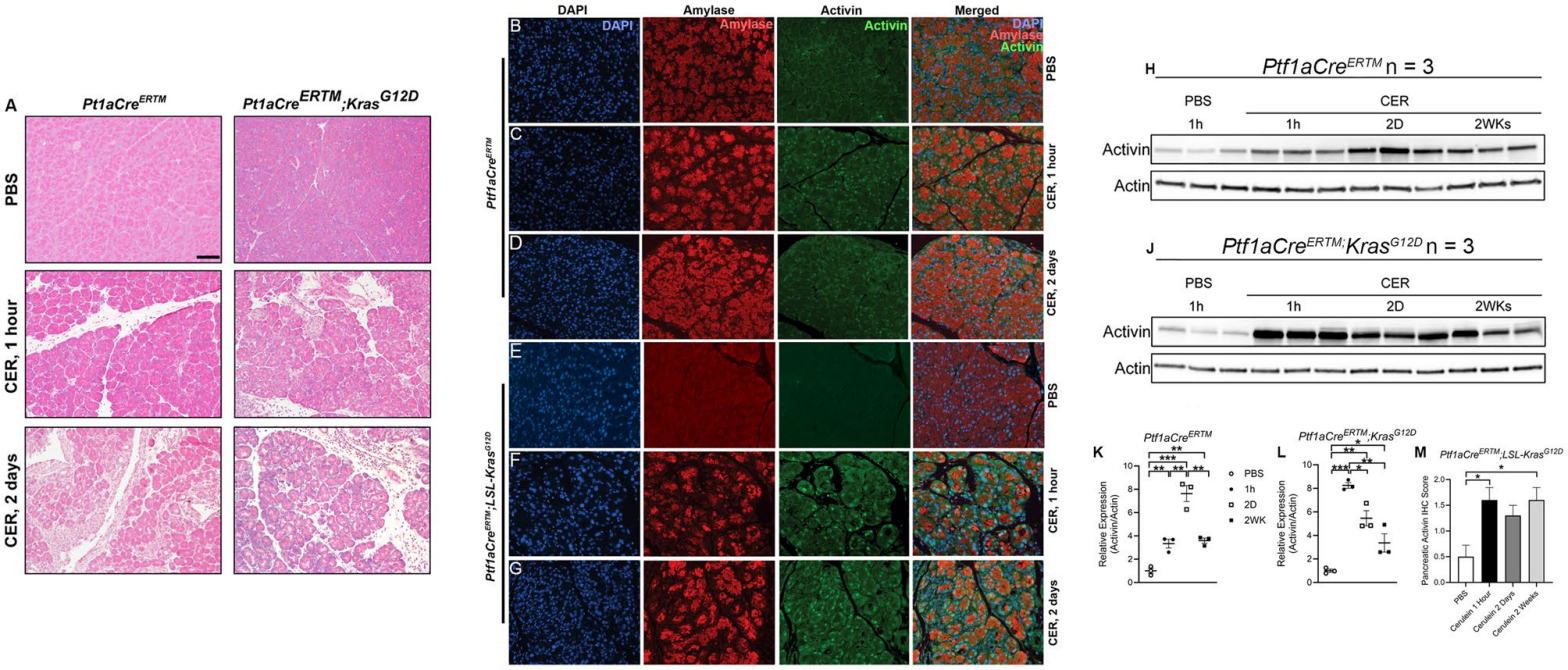
Activin production is increased in the pancreatitis. (**A**) Hematoxylin and eosin stains identified that pancreatic inflammation was achieved in the functional WT mice and which was exacerbated in the pancreatic *Kras* expressing mice. Immunofluorescent images revealed pancreatic activin production at (**B**) baseline, (**C**) 1 h following the final cerulein injection, and (**D**) two days after the final cerulein injection in functional WT *Ptf1aCre*^*ER*^*™*. Transgenic mice which express pancreatic *Kras* displayed (**E**) baseline activin expression when receiving PBS alone. These mice exhibited the greatest amount of pancreatic activin production at (**F**) 1 h and (**G**) two days following the final cerulein injection. (**H**) Western blot confirmed the increase in activin production at (**J**) 1 h, 2 days, and 2 weeks after the final cerulein injection when compared to PBS controls in *Ptf1aCre*^*ER*^*™* mice, (**K**) which was further confirmed via quantification of the western blots performed on the *Ptf1aCre*^*ER*^*™* mice, (**L**) and the *Ptf1aCre*^*ER*^*™;Kras*^*G12D*^ mice. (**M**) IHC scoring of pancreatic tissue confirmed the increase in activin production in *Ptf1aCre*^*ER*^*™;LSL-Kras*^*G12D*^ mice at each time point. Quantified western blot data analyzed via two-tailed, unpaired student’s t-test (***p < 0.001, **p < 0.01, *p < 0.05, n = 3). IHC data was analyzed via regular, one-way ANOVA with Tukey multiple comparisons test (*p < 0.05, n = 5 or 6). Representative IHC images can be found in Supplementary Fig. 1. Full and original western blot images can be found in Supplementary Fig. 2.

We previously reported that serum levels of activin correlate with disease severity in human AP and that anti-activin intervention in cerulein-induced AP in mice reduces local inflammation and disease severity^27^. We also found that anti-activin treatment reduces mortality in a more severe model of AP in which o*b/ob* mice receive IL-12/IL-18 injections^28^. To investigate the potential role for activin in both AP and inflammation-assisted PDAC development, we employed a standard AP model of cerulein oligopeptide, which produces local inflammation and mild AP^38^ in a line of transgenic mice that express the oncogenic *Kras* gene exclusively in the pancreas to further augment inflammation and induce PDAC^39^. First, we performed an H&E stain to demonstrate changes to the pancreas morphology upon cerulein treatment which were exacerbated in the pancreatic *Kras* expressing mice (Fig. 5A). Next, we performed immunofluorescent stains of the pancreatic tissue from these mice and observed baseline activin production in the pancreas of functional WT *Ptf1aCre*^*ER*^*™* mice that received PBS injections (Fig. 5B). Increased activin production in the pancreas was observed as early as 1 h following the last cerulein injection (Fig. 5C), which was maintained two days following the final cerulein injection (Fig. 5D) in the functional WT mice. Baseline expression was also observed in the transgenic mice, which exclusively express *Kras* in the pancreatic acinar cells (*Ptf1aCre*^*ER*^*™;LSL-Kras*^*G12D*^) (Fig. 5E). However, when challenged with cerulein, the most significant increase in pancreatic activin production was observed at 1 h (Fig. 5F) and two days (Fig. 5G) following the final cerulein injection. Additionally, activin co-localized with pancreatic acinar cells, indicating that these cells produce activin in response to pancreatitis early in the disease, which is maintained for at least 48 h.

To confirm that activin production increases in murine models of pancreatitis, we performed western blots on pancreatic tissue obtained from the functional WT *Ptf1aCre*^*ER*^*™* mice for activin and actin as a loading control. We observed increased activin production at 1 h, 2 days, and 2 weeks following the final cerulein injection (Fig. 5H). We then performed western blot on the transgenic pancreatic *Kras*-expressing mice. We found an even more significant increase in local activin production at 1 h, 2 days, and 2 weeks following the final cerulein injection (Fig. 5J). We then performed densitometry measurements on the western blots to quantify and confirm our findings. We observed a significant increase in relative expression of activin/actin at 1 h (3.34 relative expression units ± 0.37 S.E.M., n = 3), two days (7.63 relative expression units ± 0.69 S.E.M., n = 3), and 2 weeks (3.59 relative expression units ± 0.22 S.E.M., n = 3) following the final cerulein injection in the *Ptf1aCre*^*ER*^*™* mice when compared to PBS treated mice (1.00 relative expression units ± 0.22 S.E.M., n = 3) (Fig. 5K). This data also confirmed that the most significant increase in activin production in these mice was observed one day after the final cerulein injection. Interestingly, the most significant increase in activin production was observed at the earliest time point of 1 h (8.25 relative expression units ± 0.24 S.E.M., n = 3) following the final cerulein injection in the *Ptf1aCre*^*ER*^*™;Kras*^*G12D*^ mice. The significant increase in activin production was also maintained at two days (5.46 relative expression units ± 0.64 S.E.M., n = 3) and 2 weeks (3.36 relative expression units ± 0.77 S.E.M., n = 3) following the final cerulein injection when compared to PBS treated (1.00 relative expression units ± 0.13 S.E.M., n = 3) *Ptf1aCre*^*ER*^*™;Kras*^*G12D*^ mice (Fig. 5L). We then performed IHC scoring on pancreatic tissue sections and found a significant increase in pancreatic activin expression at 1 h (1.60 scoring units ± 0.24 S.E.M., n = 5) and 2 weeks (1.60 scoring units ± 0.24 S.E.M., n = 5) when compared to PBS treated controls (0.50 scoring units ± 0.22 S.E.M., n = 6) and no significant difference was observed at 2 days (1.30 scoring units ± 0.20 S.E.M., n = 5) (Fig. 5M). Representative IHC images can be found in Supplementary Fig. 1. In summary, activin production is increased as early as 1 h in response to pancreatic insult and is maintained up to 2 weeks following the insult.

### Chronic cerulein administration induces PDAC in pancreatic kras expressing mice

**Figure 6:**
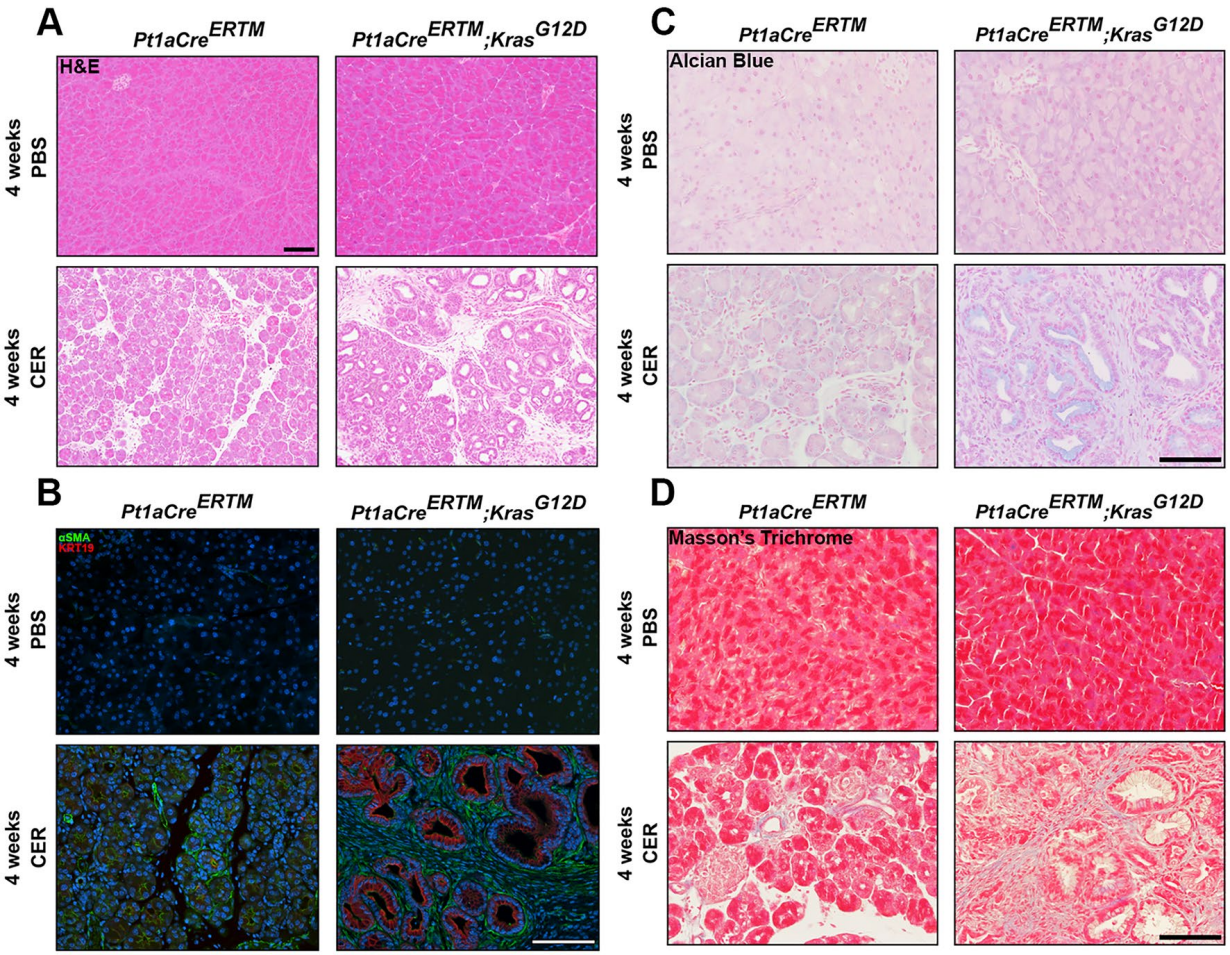
Chronic administration of cerulein to pancreatic expressing *Kras* mice promotes fibroblast activation and progresses to early pancreatic neoplasia. (**A**) Hematoxylin and Eosin (H&E) staining confirmed that cerulein alone induced mild inflammation in functional WT (*Ptf1aCre*^*ER*^*™*) mice which progressed to early pancreatic neoplasia in pancreatic *Kras* expressing *Ptf1aCre*^*ER*^*™;LSL-Kras*^*G12D*^ mice. (**B**) Immunofluorescent staining for α-smooth muscle actin (αSMA) revealed significant fibroblast activation in functional WT mice which was further exacerbated and coupled to increased KRT19 expression in pancreatic *Kras* expressing mice. (**C**) Alcian Blue staining was increased in the pancreatic *Kras* expressing mice exclusively. (**D**) Masson’s Trichrome staining revealed increased collagen deposits in functional WT mice receiving chronic administrations of cerulein which was exacerbated in pancreatic *Kras* expressing mice.

To confirm that chronic cerulein administration progresses towards early pancreatic neoplasia in pancreatic *Kras-*expressing mice, we performed several stains on pancreatic tissue to look for evidence of neoplastic lesions. We first performed a Hematoxylin and Eosin (H&E) stain, revealeing significant inflammatory infiltrate and mild pancreatic damage which was exacerbated in the pancreatic *Kras*-expressing mice. Furthermore, several pancreatic intraepithelial neoplastic (PanIN) lesions were observed throughout the pancreas of pancreatic *Kras*-expressing mice that received cerulein (Fig. 6A). To confirm the development of fibrotic tissue within the PanINs, we next performed immunofluorescent staining for the fibroblast activation protein α-smooth muscle actin (αSMA) and the cancer cell marker Keratin-19 (KRT19). Functional WT mice that received cerulein displayed an increase in αSMA, also observed in the pancreatic *Kras-*expressing mice that received cerulein. However, several KRT19^+^ PanIN lesions surrounded by the fibrotic environment were observed in the pancreatic *Kras-*expressing mice that received cerulein compared to control mice (Fig. 6B). Metaplastic lesions in the pancreas often express Alcian Blue^+^ mucins^40^. Therefore, we also performed Alcian Blue staining and found several Alcian Blue^+^ mucins in the PanIN lesions of the pancreatic *Kras*-expressing mice that received cerulein, which was not observed in any other condition (Fig. 6C). Masson’s Trichrome staining revealed mild collagen deposition in the functional WT mice that received cerulein exacerbated in the pancreatic *Kras-*expressing mice (Fig. 6D). These stains confirmed that we successfully generated a model of CP in our functional WT mice, which progressed to early pancreatic neoplasia in pancreatic *Kras*-expressing mice.

### Increased activin production is maintained in CP and is further increased in chronic inflammation-assisted PDAC development

**Figure 7:**
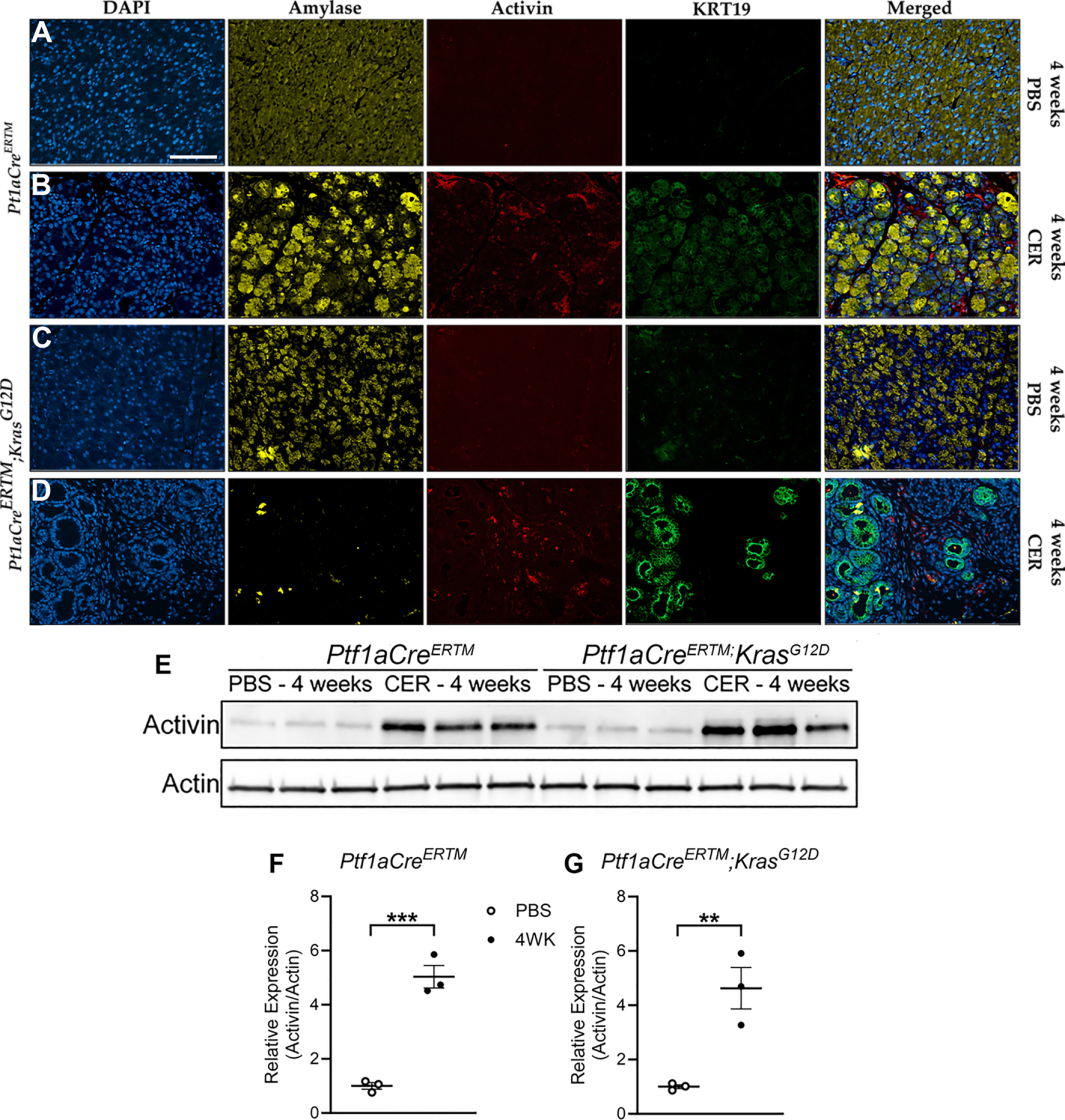
Increased activin production is maintained throughout chronic inflammation-assisted PDAC development and co-localizes with cells undergoing acinar-to-ductal metaplasia. Immunofluorescent imaging revealed (**A**) baseline activin expression in healthy pancreatic tissue and (**B**) increased activin and KRT19 levels in CP treated *Ptf1aCre*^*ER*^*™* mice. (**C**) Baseline expression of activin was maintained 4 weeks following pancreatic *Kras* induction, (**D**) which was increased and co-localized with Krt-19 in pancreatic *Kras*-expressing mice in the CP model and (**E**) was further confirmed via quantification of the western blots performed on *Ptf1aCre*^*ER*^*™* mice and (**F**) *Ptf1aCre*^*ER*^*™;Kras*^*G12D*^ mice. Data analyzed via two-tailed, unpaired student’s t-test (***p < 0.001, **p < 0.01, n = 3). Full and original western blot images can be found in Supplementary Fig. 3.

To test the hypothesis that activin production is maintained throughout the development of inflammation-assisted PDAC in a chronic setting, we performed cerulein injections for 4 weeks and performed an immunofluorescent stain for DAPI, activin, Amylase, and KRT19 as a marker for acinar-to-ductal metaplasia (ADM) which occurs as a result of pancreatic *Kras* expression^41^. We observed reduced baseline activin production in the PBS-treated mice regardless of *Kras* expression (Fig. 7A,C). Increased activin production was observed in the functional WT mice exposed to the chronic cerulein injections (Fig. 7B). The most significant amount of activin production was observed in the pancreatic *Kras*-expressing mice exposed to the chronic cerulein administrations (Fig. 7D). Interestingly, activin co-localization was observed exclusively in KRT19-expressing cells, suggesting activin plays a significant role in mediating ADM progression toward PDAC. The increased pancreatic activin expression was further confirmed via western blot (Fig. 7E). Densitometry was performed on the western blots to quantify our findings. These data confirmed that there was a significant increase in relative activin/actin production at 4 weeks following the final cerulein injection in *Ptf1aCre*^*ER*^*™* mice (5.04 relative expression units ± 0.42 S.E.M., n = 3) when compared to PBS-treated controls (1.00 relative scoring units ± 0.12 S.E.M., n = 3) (Fig. 7F). This effect was conserved in the *Ptf1aCre*^*ER*^*™; Kras*^*G12D*^ mice when comparing activin production at 4 weeks following the final cerulein injection (4.63 relative expression units ± 0.76 S.E.M., n = 3) and PBS-treated controls (1.00 relative expression units ± 0.07 S.E.M., n = 3) (Fig. 7G). These data suggest that increased pancreatic activin production is maintained during PDAC development.

## Discussion

One unique challenge to AP research is the inability to obtain pancreatic biopsy samples from human AP patients. Surgical intervention is not recommended in mild and moderate AP, and the World Society of Emergency Surgery (WSES, 2019) guidelines only recommend surgery for drainage or in severe bleeding^42,43^. Furthermore, the WSES recommends deferring surgical intervention until 4 weeks following the initial stage to permit better differentiation and removal of necrotic tissue^43^. AP typically resolves within the first week^44^; therefore, obtaining tissue samples four weeks following disease onset will not provide insight into the cellular mechanisms driving severe disease. Our group obtained serial sections of one chronic pancreatitis tissue sample with fibrosis and PanIN lesions; ROIs were specifically selected in regions of the tissue where PanCK was not visualized to avoid regions of PanIN lesions and ADM. Although we could not obtain a tissue section from an AP patient, the data obtained from our DSP analysis confirmed that activin co-localization is associated with immune cell activation in pancreatitis, likely occurring in AP. Despite its limitations, this research holds the potential to significantly impact patient care, providing a deeper understanding of the disease mechanisms and potential therapeutic targets.

Despite their shortcomings, the field must rely heavily on animal models to investigate the mechanisms driving AP and identify novel therapeutic targets. Choline-deficient ethionine-enriched (CDE) diet-based models are only effective in females, providing a significant gap in translatability as no effect of gender is observed in human AP^45,46^. Surgical sham operations have been shown to increase activin levels^47^. Therefore, surgical models were not considered for these studies. We previously published data employing the *ob/ob* IL-12/IL-18 mouse model, which yielded robust results. However, we also observed serum activin levels correlated with disease severity in humans independent of BMI^27,28^. Therefore, we wanted to utilize a separate model in which obesity was not a confounding variable.

We have chosen to employ the well-described cerulein-based model which induces mild inflammation in functional WT mice, providing a suitable model for identifying the role for activin early in response to pancreatic inflammation^28,48^. Inducible, tissue-specific *Kras* models create a proinflammatory milieu within the pancreas, which cerulein injections can then augment to induce PDAC development^48^. The relationship between *Kras* mutations and inflammation and notably, between *Kras* mutations and spontaneous induction of ADM have been reported^49–54^. Thus, to identify the role of activin in inflammation-assisted PDAC development, we employed the well-described inducible animal model^39,55–57^ and confirmed that activin production is increased in pancreatitis induced in functional WT mice which was further exacerbated in the presence of *Kras*. These data suggest that activin may be a therapeutic target for AP, CP, and early PDAC development in humans. Furthermore, anti-activin intervention is well tolerated in humans^58^, providing an attractive target for therapeutics for AP and CP patients.

Recently, a five-cytokine panel including angiopoietin-2 (Ang-2), Hepatocyte Growth Factor (HGF), IL-8, resistin, and TNF-receptor superfamily IA was developed, which accurately predicts persistent organ failure in AP with a tenfold cross-validated accuracy of 0.89^59^. The success of this panel suggests that therapeutic strategies focused on circulating cytokines may benefit AP patients since these molecules are targetable and upregulated early in the disease. Interestingly, activin appears to interact with several members of this cytokine panel. Indeed, TNFα stimulates neutrophils to release activin, leading to increased TNFα production^31^, activin stimulates IL-8 production in endometrial stromal cells^60^, and inhibition of the SMAD protein downstream of activin increases Ang-2 production^61^, suggesting activin signaling may result in stimulation of inflammatory cells and reduction of vascularization. Additionally, activin stimulates apoptosis of hepatocytes, and increased levels of circulating activin are observed in severe alcoholic hepatitis patients, suggesting that activin exerts direct effects on liver function, likely altering HGF production^62,63^. More research is required to determine if a relationship between activin and resistin exists.

Macrophages are a major innate immune cell type that can release and secrete enzymes, cytokines, and several other compounds upon stimulation^64^. RAW264.7 macrophages can produce several inflammatory cytokines including, IL-1β, TNF-α, and IL-6, upon stimulation^65^, which mimics what is observed in primary macrophages^66^. Macrophages are the most abundant inflammatory cell type found in the inflamed pancreas in AP^67,68^. Identifying pathways that stimulate or suppress macrophage activity in AP and CP is critical to advancing available therapeutic options for these patients. Activation of the MAPK pathway occurs early in AP and inhibition of this pathway has been shown to alleviate inflammation in AP-induced acute lung injury^69,70^. Our data suggest that activin may be responsible for the activation of the MAPK pathway in macrophages which promotes their infiltration into the pancreas.

Neutrophils are the most abundant white blood cells in circulation and are essential effector cells of the innate immune system^71^. Depletion of neutrophils ameliorates disease progression in several mouse models of AP^72,73^. Administration of the pro-inflammatory molecule tumor necrosis factor-α to neutrophils stimulates their production of activin^31^ suggesting a pro-inflammatory role for activin in these cells. Therefore, we investigated the ability of activin to stimulate neutrophil activation and found that activin is associated with increased expression of the neutrophil activation marker CD66b in situ. We also found that direct stimulation of DMSO-differentiated HL-60 cells with activin increases the production of the mRNA necessary for CD66b expression. Similar to what has been previously reported, we did not see an increase in the mRNA necessary for CD11b expression following 24 h of DMSO stimulation^74^. Interestingly, we did observe an increase in mRNA production for CD11b following activin stimulation regardless of DMSO exposure, suggesting that activin may increase expression of the migratory marker CD11b in immature neutrophils. Future experiments will determine if this increase in CD11b is associated with the increased migratory capacity of these cells.

Improving the treatment of AP, CP, and PDAC is essential to reducing population disease burdens and increasing overall survival rates in pancreatic cancer. The data presented here provides compelling evidence that (i) pancreatic activin production is increased as early as 1 h into pancreatitis and is maintained throughout CP progression, (ii) activin stimulates macrophage migration and neutrophil activation, and (iii) activin production is further increased in the pancreas in a mouse model of inflammation-assisted PDAC development. Together, these experiments demonstrate that activin is an early, targetable molecule that perpetuates AP's inflammatory response, promotes ADM in CP, and mediates the development of aggressive PanIN lesions. Activin inhibition is well tolerated in humans^58^; therefore design of a clinical trial of activin inhibition in AP patients may allow for rapid translation into impactful clinical application.

## Materials and methods

### Reagents and antibodies

All methods were performed in accordance with the relevant guidelines and regulations. Activin was reconstituted in PBS containing 4 mM HCl according to the manufacturer’s instructions (both R&D, Minneapolis, MN, USA). Tamoxifen (Sigma-Aldrich, Cat. # T5648) and cerulein (BACHEM, Cat. # H3220) were purchased from their manufacturers. Anti-activin neutralizing antibody (AF388, R&D Systems) was purchased from the manufacturer. Inhibitor U0126 was reconstituted in DMSO according to the instructions from the manufacturer (EMD Millipore Corp. Burlington, MA, USA). Tamoxifen was solubilized via sonication in corn oil (Sigma-Aldrich, Cat. # C8267) for in vivo experiments. Antibodies for β-actin (Sigma-Aldrich, Cat. # A1978) and activin (Inhibin-βA, Ansh Labs, Webster, TX, USA, Cat. # AB-305-A1063, the same antibody was used for western blot, IHC, and IF) were obtained from the manufacturer for western blot. Immunofluorescent antibodies KRT19 (DSHB, Iowa City, IA, USA, Cat. # TROMA-III), Amylase (Santa Cruz Biotechnology, Dallas, TX, USA, Cat. # sc-46657), Activin A (Ansh Labs, Webster, TX, USA, Cat. # AB-305-A1063), and αSMA (Abcam, Boston, MA, USA, Cat. # ab5694), were used as primary antibodies and Alexa Fluor-labeled secondary antibodies (goat anti-rabbit 488, goat anti-mouse 594, and goat anti-rat AF 647, ThermoFisher Scientific, Cat. # A11008 and Jackson Immunoresearch, West Grove, PA, USA, Cat. #115-585-003 and # 112-175-167, respectively) were also employed for immunofluorescence.

### Mouse experiments

Stony Brook University Institutional Animal Care and Use Committee approved all animal experiments which comply with ARRIVE guidelines. The animal study protocol was approved by the Institutional Animal Care and Use Committee (IACUC) of Stony Brook University (protocol code 1142963 and date of approval of 10/19/2021). All methods were performed in accordance with the relevant guidelines and regulations. *Ptf1a-Cre*^*ER*^*™* (Jackson Laboratory, Bar Harbor, Maine; Stock number 091378) and *LSL-Kras*^*G12D*^ (Jackson Laboratory, Stock Number: 008179) were cross-bred to generate *Ptf1aCre-*^*ER*^*™;LSL-Kras*^*G12D*^ as previously described^39^. All mice received three injections of tamoxifen (3 mg/dose) dissolved in corn oil or corn oil control intraperitoneally (i.p.) on alternating days as previously described^39^. Acute pancreatitis was induced forty-eight h following the final tamoxifen injection, mice received hourly i.p. injections of 100 µL of cerulein (50 µg/mL) dissolved in PBS for 6 h on two consecutive days^39^. Pancreatic tissues were collected 1 h and 2 days after the last cerulein injection. For chronic pancreatitis, forty-eight h after the final tamoxifen injection, mice received hourly i.p. injections of 100 µL of cerulein (50 µg/mL) dissolved in PBS for 6 h on 3 days a week for 3 weeks^75^. At the end of the treatment mice were euthanized and pancreatic tissues were collected. The tissues from both protocols were collected in 10% neutral buffered formalin and transfer to 70% ethanol within 48 h. The fixation, embedding, and sectioning of the tissues were performed by the Research Histology Core at Stony Brook University.

### Digital spatial profiling

The DSP assay (NanoString Technologies, Seattle, WA, USA) was performed as previously described^76,77^. Briefly, one slide containing one human chronic pancreatitis patient sample was obtained under the IRB protocol IRB2022-00587. The study was conducted in accordance with the Declaration of Helsinki, and approved by the Institutional Review Board of Stony Brook University (protocol code IRB2022-00587 and date of approval of 11/29/2022). All methods were performed in accordance with the relevant guidelines and regulations. This slide was used to select 26 ROIs where PanCK expression was not co-localized and then were separated into 52 AOIs (26 activin ( +) and 26 activin (−)) for collection and analysis. Collected barcodes were hybridized and quantified using the nCounter Sprint Profiler as previously described^78^. Data QC was performed as previously described^77^. The geometric mean expression of the housekeeping proteins S6 and Histone H3 were used for data normalization based upon the correlation of their expression across all 52 samples that passed QC (data not shown). Data expressed as normalized counts.

### Transwell migration assay and RAW264.7 cell culture

RAW264.7 mouse-derived macrophages were a gift from Dr. Jun Sun (University of Illinois, Chicago, USA) and were maintained in DMEM (Corning, Corning, NY, USA) supplemented with 10% fetal bovine serum (FBS) at 5% CO_2_ and 37 °C. Fibronectin (2 μg/mL; Sigma-Aldrich, St. Louis, MO, USA) was used as a chemoattractant, and 1 × 10^5^ cells (RAW264.7 cells or HL-60 cells) were permitted to migrate for 6 h following stimulation as previously described^79^. Migrated cells were stained with DAPI, imaged on an Evos FL Auto 2D, and five microscopic fields of each well were taken and manually counted (ThermoFisher Scientific, Waltham, MA, USA). Each n-value represents the average count of one transwell. Experiments were performed in duplicate or triplicate and were repeated four times to ensure that the observed effects were conserved across passages.

### HL-60 cell culture, differentiation, and stimulation

1 × 10^5^ cells/mL HL-60 cells were seeded in complete RPMI media (Corning, Corning, NY, USA) and maintained at 37 °C, 5% CO_2_. To stimulate differentiation into neutrophil-like cells as previously described^37^, 1.25% DMSO was added to the cell solution for 24 h followed by a 24-h stimulation with activin in FBS-free media.

### RNA isolation and quantitative real-time PCR (qPCR)

A RNeasy kit (Qiagen, Valencia, CA, USA) was used to isolate total RNA from HL-60 cells and first-strand cDNA was synthesized using a SuperScript VILO cDNA Synthesis Kit (ThermoFisher Scientific, Waltham, MA, USA). PowerTrack SYBR Green Master Mix (ThermoFisher Scientific) was employed with primers for *Gapdh*, *Ceacam8* (*CD66b*), and *ITGAM* (*CD11b*; all Integrated DNA Technologies, Coralville, IA, USA). qPCR reactions were performed and quantified on a Lightcycler 480 II (Roche Diagnostics, Seattle, WA, USA). Differences between DMSO- and/or activin-treated HL-60 cells were confirmed via the common delta-delta (2^−ΔΔCq^) method with *Gapdh* as the housekeeping gene^80^.

### Cell viability assay

The Cell Counting Kit-8 (CCK-8, Dojindo Technologies, Rockville, MD, USA) was employed for a cell viability assay on the RAW264.7 macrophages exposed to the same conditions as the transwell migration assay. 5 × 10^3^ cells/well were seeded into a 96-well plate in the presence/absence of vehicle (4 mM HCl in 1 × PBS), activin, anti-activin, and U0126. The CCK-8 solution was added to each well and the plate was incubated for 6 h prior to quantification via optical density measurement at 450 nm.

### Immunofluorescent imaging

Immunofluorescence was performed as previously described^81^. Briefly, slides were deparaffinized and rehydrated before antigen retrieval and overnight primary antibody incubation. Secondary antibodies were incubated for 30 min at 37 °C, counterstained with Hoechst 33,258 (ThermoFisher Scientific, Cat. # H3569), and slides were mounted with Fluoromount Aqueous Mounting Medium (Millipore-Sigma, Cat. F4680). Fluorescent images were captured and analyzed using Nikon Eclipse 90i microscope (Nikon).

### Western blot

Total protein from pancreatic lysates was extracted from cells with Laemmli buffer, and the analysis was performed as previously described using the antibodies mentioned above^82^. Actin was used as a loading control (Sigma-Aldrich, St. Louis, MO, USA, Cat.# A1978).

### Immunohistochemistry, H&E, Masson’s trichrome, and Alcian blue staining

Immunohistochemical staining (IHC) of activin was performed as previously described^83^. Slides were blindly scored by two independent investigators and received scores of 0 (no staining), 1 (intermediate staining), or 2 (high staining). Hematoxylin and eosin staining was performed as previously described^84^ on a serial section of the human CP tissue section obtained for DSP analysis (IRB protocol IRB2022-00,587) and sections obtained from mouse tissues. Alcian Blue and Masson’s Trichrome staining were performed as previously described^39,75^. All imaging was performed on an Evos FL Auto 2D microscope.

### Statistical analysis

Linear mixed modeling (LMM) was performed on all DSP data with the Benjamin–Hochberg correction test as previously described^77,85^ (NanoString DSP Analysis Suite Software version 2.5.1.145). Data obtained from the LMM analysis were used to generate the heatmap included in Fig. 2 using Rstudio 2022.07.02 and the package “gplots” (**p < 0.01, ***p < 0.001, ****p < 0.0001). Data in Fig. 4 were analyzed via regular one-way ANOVA, post-hoc analysis performed via Tukey’s multiple-comparisons test (GraphPad Prism 10 software, **p < 0.01). IHC scoring data in Fig. 5 and western blot data in Fig. 6 were both analyzed via ordinary, one-way ANOVA (GraphPad Prism 10 software, **p < 0.01, ****p < 0.0001).

### Supplementary Information

Supplementary Information.

## Data Availability

All relevant data is included in the manuscript or in the supplementary materials.
