# Supplementary material for: Activin A signaling stimulates neutrophil activation and macrophage migration in pancreatitis
Source: Sci Rep. 2024 Apr 23;14:9382. doi: 10.1038/s41598-024-60065-y (PMC11039671; doi:10.1038/s41598-024-60065-y)
Supplement: Supplementary file 1 — Supplementary Information. [file 41598_2024_60065_MOESM1_ESM.docx]

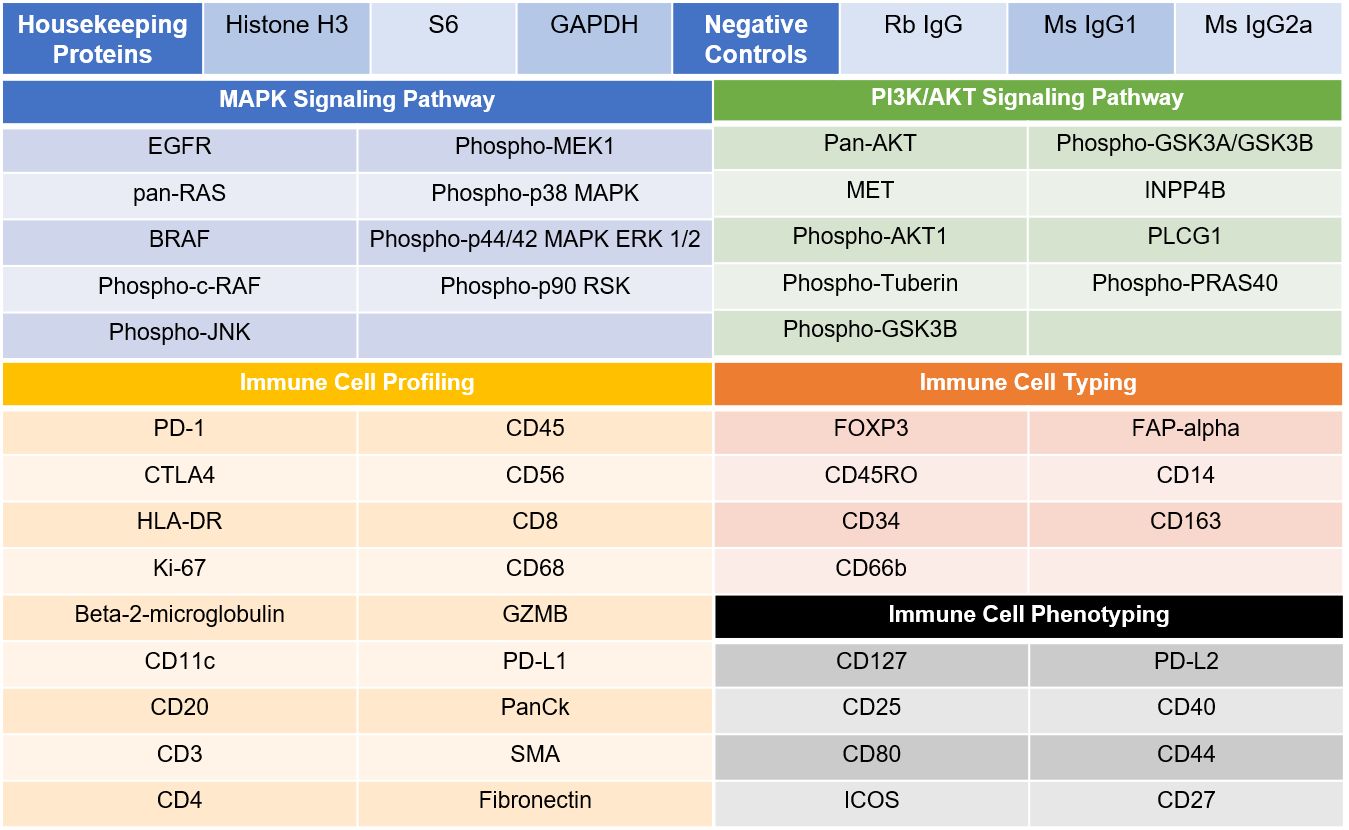
**Supplemental Table 1: List of the 57 quantitative markers employed in the DSP experiments including both positive and negative controls which were used for normalization.**


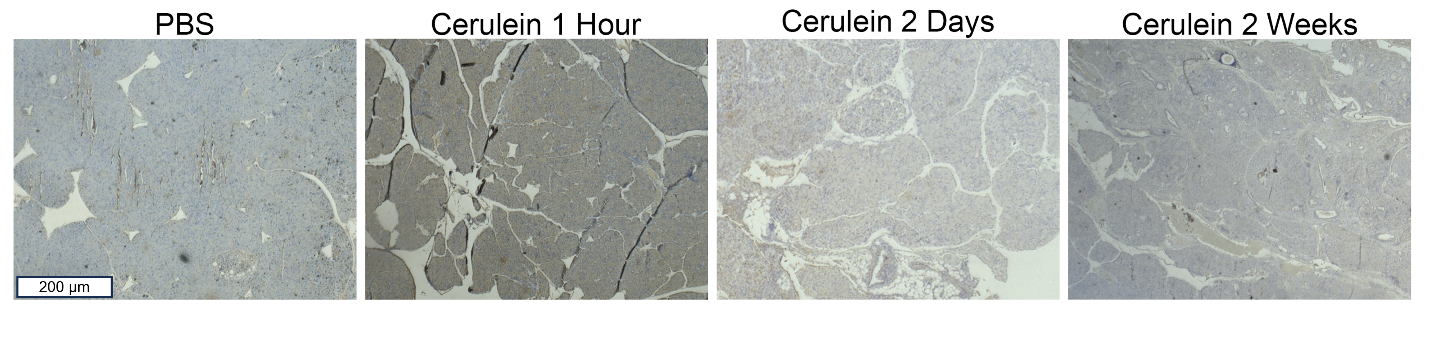


**Supplemental Figure 1: Representative IHC images used to provide the scoring data in Figure 5K.** The following scores were assigned to these specific representative images: PBS 0; 1 Hour 2; 2 Days 1.5; 2 weeks 1.


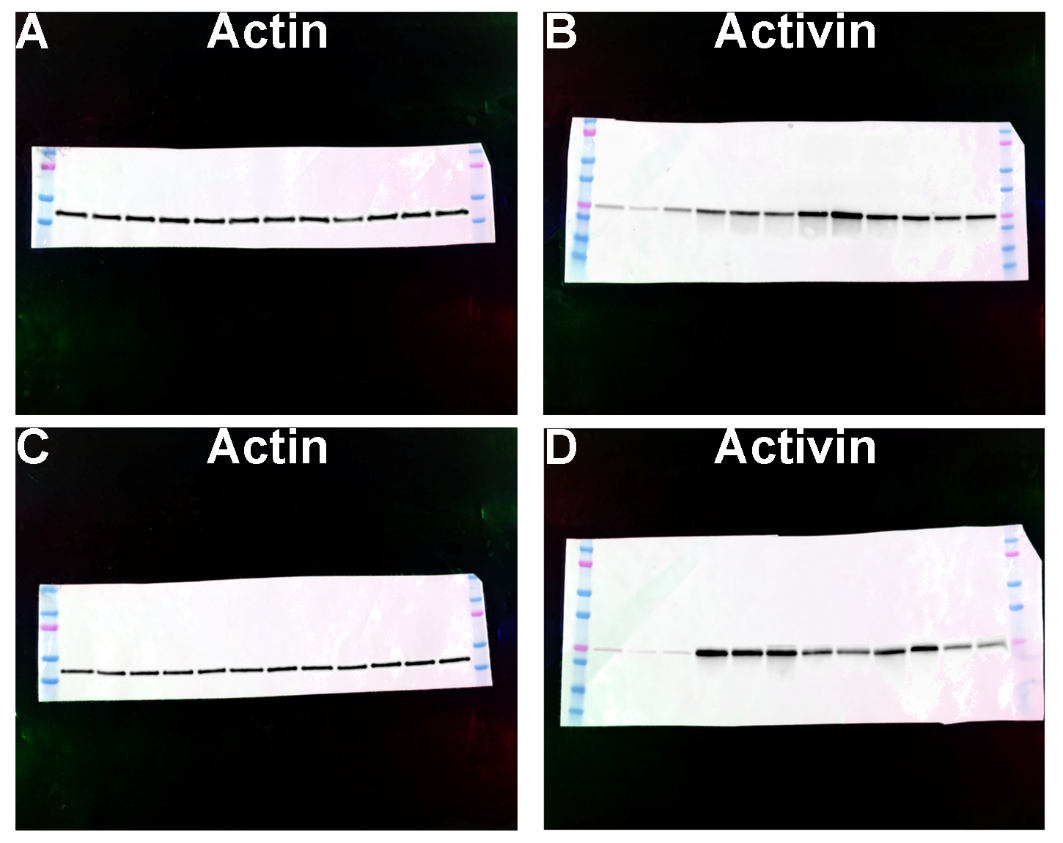


**Supplemental Figure 2: Original western blot images that were used for Figure 5 in the mouse model of AP.**

**
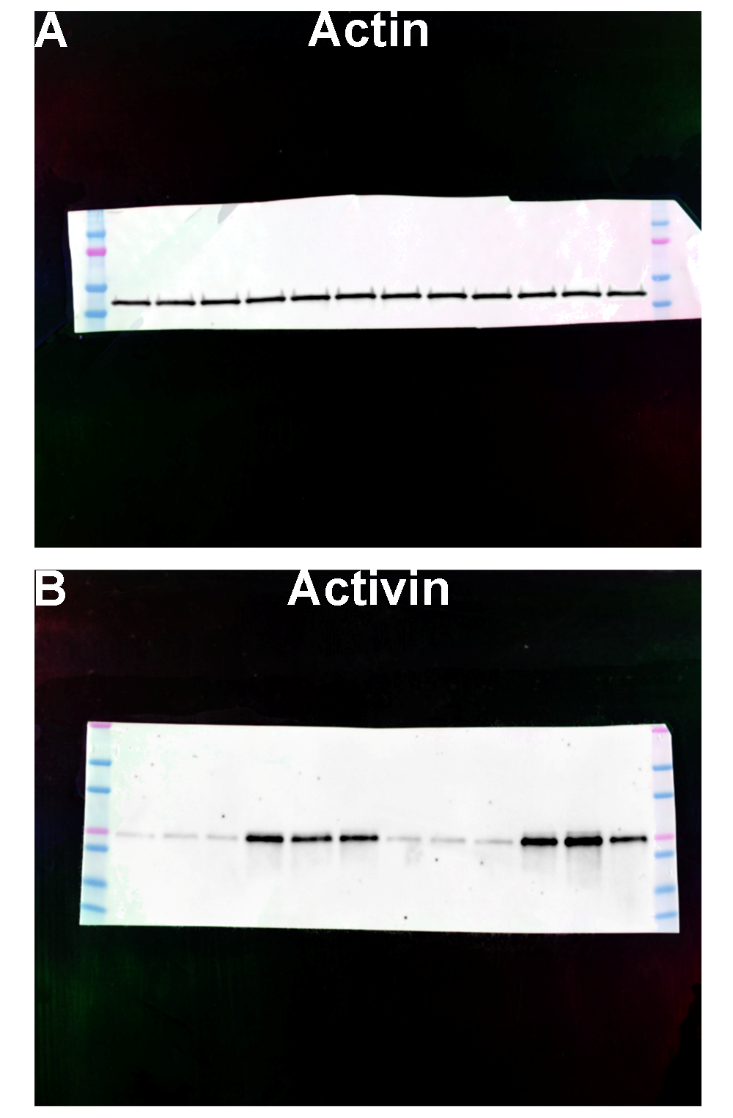
**

**Supplemental Figure 3: Original western blot images that were used for Figure 7 in the mouse model of CP.**
